# Prevalence of Depression and Anxiety Disorders and Associated Psychosocial Risk Factors Among Pregnant Women Attending an Outpatient Clinic at a Tertiary Care Hospital

**DOI:** 10.7759/cureus.103511

**Published:** 2026-02-12

**Authors:** Pavithira Annamalai, Rohit Balakrishnan, Pavithra Devi Babu

**Affiliations:** 1 Psychiatry, Pondicherry Institute of Medical Sciences, Puducherry, IND; 2 Obstetrics and Gynaecology, Pondicherry Institute of Medical Sciences, Puducherry, IND

**Keywords:** anxiety, health facility, peri-partum depression, pregnant women, psychosocial factors

## Abstract

Background

Pregnancy is a period of significant biological and psychosocial change that may predispose women to mental health disorders. Antenatal depression and anxiety are increasingly recognized as important public health concerns due to their adverse impact on maternal well-being, pregnancy outcomes, and offspring development. Despite this, these conditions often remain underdiagnosed in routine antenatal care, particularly in low- and middle-income settings. This study aimed to determine the prevalence of depressive and anxiety disorders among pregnant women attending a tertiary care outpatient clinic and to examine their association with selected sociodemographic, obstetric, psychosocial, and physiological factors.

Methods

A hospital-based cross-sectional study was conducted over two months in the obstetrics and gynaecology outpatient department of a tertiary care teaching hospital. A total of 180 antenatal women aged ≥18 years were enrolled. Data were collected using a semi-structured questionnaire covering sociodemographic details, obstetric history, psychosocial stressors, and physiological parameters. Depression and anxiety disorders were diagnosed using the Structured Clinical Interview for DSM-5 (SCID-5). Descriptive statistics were used to estimate prevalence, and bivariate and multivariable logistic regression analyses were performed to identify factors associated with depression and anxiety.

Results

Most were aged ≥25 years, 171 (95.0%), with more than half of the women being graduates, 105 (58.3%), and the majority were homemakers, 154 (85.6%). The prevalence of prenatal depression and anxiety was 31(17.2%) and 14(7.8%), respectively. On multivariable analysis, depression was independently associated with duration of marriage greater than five years (18/69, 26.1%; adjusted odds ratio (AOR) = 2.47, 95% CI: 1.10-5.55), unplanned pregnancy (9/24, 37.5%; AOR = 2.9, 95% CI: 1.1-7.8), presence of comorbidities (25/80, 31.3%; AOR = 4.8, 95% CI: 1.7-13.6), economic stress (20/31, 64.5%; AOR = 9.6, 95% CI: 3.6-25.4), work-related stress (18/27, 66.7%; AOR = 8.2, 95% CI: 3.1-21.6), and marital discord (19/25, 76.0%; AOR = 14.7, 95% CI: 4.9-43.8). Anxiety was independently associated with unplanned pregnancy (10/24, 41.7%; AOR = 9.8, 95% CI: 2.9-33.1), economic stress (9/30, 30.0%; AOR = 5.4, 95% CI: 1.6-18.3), and marital discord (6/25, 24.0%; AOR = 3.4, 95% CI: 1.0-11.9).

Conclusion

A substantial proportion of pregnant women experience depression and anxiety, largely driven by psychosocial stressors and certain obstetric factors. Integrating routine mental health screening and psychosocial support into antenatal care services is essential to improve maternal mental health and pregnancy outcomes.

## Introduction

Pregnancy is traditionally regarded as a normal and positive psychosocial transition in a woman’s life; however, growing evidence suggests that it is also a period of heightened vulnerability to mental health problems. Profound hormonal, physiological, emotional, and social changes occur during pregnancy, and for a substantial proportion of women, these changes may precipitate or exacerbate psychological distress, depression, and anxiety. When unrecognized or untreated, antenatal mental health disorders can adversely affect both the mother and the developing foetus, with consequences extending beyond pregnancy into the postpartum period and childhood. Adverse pregnancy outcomes associated with antenatal depression and anxiety (perinatal depression and anxiety) are a mood disorder characterized by clinically significant depressive/anxiety symptoms that occur during pregnancy (antenatal period) and/or within the first year after childbirth (postpartum period). include low birth weight, prolonged labour, preterm delivery, intrauterine growth restriction, and an increased likelihood of caesarean section [1].

In recent years, perinatal mental health has gained global attention as an essential component of maternal healthcare. The World Health Organization (WHO), in its guidance on integrating perinatal mental health into maternal and child health services, highlights that perinatal depression and anxiety are common worldwide [2]. It is estimated that approximately one in ten women in high-income countries and nearly one in five women in low- and middle-income countries (LMICs) experience significant mental health disorders during the perinatal period, underscoring the substantial global burden of these conditions. Evidence from recent studies indicates that the prevalence of depressive and anxiety disorders during pregnancy may be as high as 35.7% and 55.7%, respectively, reflecting a serious public health concern, particularly in LMIC settings [3].

Maternal mental health during pregnancy has important implications for foetal brain development and long-term offspring outcomes. Experimental and epidemiological studies have demonstrated that maternal stress, depression, and anxiety during pregnancy can influence foetal neurodevelopment by altering stress-response systems and brain structures involved in emotional regulation and cognitive functioning [4]. These alterations may predispose offspring to a higher risk of emotional, behavioural, and psychiatric disorders later in life, suggesting that antenatal mental health disorders have intergenerational consequences [5]. Thus, addressing maternal mental health during pregnancy is critical not only for improving maternal well-being but also for promoting optimal child development.

A wide range of psychosocial, demographic, and obstetric factors has been implicated in the increasing prevalence of depression and anxiety during pregnancy. These include maternal age, educational status, socioeconomic background, employment status, and marital circumstances, as well as obstetric factors such as parity, unplanned pregnancy, previous pregnancy loss, and current pregnancy-related complications. Psychosocial stressors such as financial insecurity, work-related stress, marital discord, lack of social and emotional support, exposure to domestic violence, and stressful life events have consistently been shown to play a pivotal role in the onset and persistence of antenatal depression and anxiety [6]. Women from lower educational and socioeconomic backgrounds may be particularly vulnerable due to limited access to healthcare resources, reduced coping mechanisms, and heightened exposure to chronic stressors [6].

Evidence from Indian and international studies further highlights the magnitude of this problem. Hospital-based studies in India have reported a high prevalence of antenatal depression, with significant associations observed with low social support, marital discord, domestic violence, and low-income status [1,7]. Similarly, studies examining pregnancy-related anxiety have reported high prevalence rates and identified lower socioeconomic status, poor social support, and coexisting depression as key determinants [8-10]. Other Indian studies using standardised screening tools have reported variable prevalence rates of anxiety disorders during pregnancy, reflecting differences in study populations, assessment tools, and sociocultural contexts [7,8].

Beyond immediate maternal outcomes, antenatal depression and anxiety may also influence obstetric decision-making and delivery outcomes. Studies have shown that women experiencing depression and anxiety during pregnancy are more likely to undergo caesarean delivery, suggesting that psychological factors may affect perceptions of childbirth, pain tolerance, and clinical decision-making [8-10]. These findings further emphasise the need for early identification and management of mental health disorders during pregnancy.

Despite the growing body of evidence, antenatal mental health remains under-recognised and under-addressed in routine obstetric care, particularly in tertiary care outpatient settings in LMICs. Systematic assessment of depression and anxiety and identification of associated psychosocial risk factors are essential for designing targeted interventions and integrating mental health services into existing maternal healthcare frameworks. This study aimed to determine the prevalence of depressive and anxiety disorders among pregnant women attending a tertiary care outpatient clinic and to assess the association between psychosocial risk factors and the occurrence of depression and anxiety during pregnancy.

## Materials and methods

Study setting

The study was conducted in the Obstetrics and Gynaecology Outpatient Department of a tertiary care teaching hospital in Puducherry. This setting caters to a large and diverse antenatal population, making it suitable for assessing the prevalence of mental health disorders and associated psychosocial risk factors among pregnant women receiving routine antenatal care.

Study design and period

A hospital-based cross-sectional study design was adopted. This design was chosen to estimate the prevalence of depression and anxiety disorders during pregnancy and to examine their association with selected psychosocial and obstetric factors at a single point in time. The study was carried out over a period of two months (June-July 2023).

Study population

The study population comprised antenatal women attending the outpatient antenatal clinics of the tertiary care hospital during the study period. Pregnant women presenting for routine antenatal check-ups constituted the source population from which eligible participants were recruited based on convenience sampling. 

Sample size estimation

The sample size was calculated using the prevalence of antenatal depression reported in a previous hospital-based study conducted in Bangalore, which documented a prevalence of 35.6% [2]. Using this prevalence estimate, a 95% confidence interval, and an absolute precision of 7%, the minimum required sample size was calculated as 180 participants. Accordingly, a final sample size of 180 antenatal women was included in the study.

Inclusion criteria

Pregnant women aged 18 years and above, with a confirmed pregnancy, attending the antenatal outpatient clinic were eligible for inclusion. Participants were required to be able to understand the nature and purpose of the study, as well as the questions and instructions provided by the research assistant. Only those who were willing to participate and provided written informed consent were enrolled. Pregnant women with obstetric or medical complications in the current pregnancy were also included, as these conditions may influence psychological well-being and were relevant to the study objectives.

Exclusion criteria

Pregnant women were excluded from the study if they had a previously diagnosed psychiatric disorder (including major depressive disorder, bipolar disorder, psychotic disorders, or anxiety disorders) and were currently receiving psychiatric treatment, as this could confound prevalence estimates. Women with cognitive impairment, intellectual disability, or severe communication difficulties that limited their ability to comprehend the study procedures or respond reliably to the assessment tools were excluded.

Data collection procedure and instruments

All antenatal women attending the Obstetrics and Gynaecology Outpatient Department during the study period were approached consecutively. Eligible participants were identified after screening for inclusion criteria and were informed about the purpose, procedures, and voluntary nature of the study. Written informed consent was obtained prior to participation. Privacy was ensured during the interview process, and confidentiality of information was maintained throughout the study.

Data were collected using a semi-structured interviewer-administered questionnaire. The questionnaire included sections on sociodemographic characteristics, obstetric history, medical and psychiatric history, and psychosocial stressors. Standardized and calibrated instruments were used to measure height and weight, which were subsequently used to calculate body mass index (BMI). Information on haemoglobin levels was obtained from hospital records to ensure accuracy and reduce participant burden.

Study measures

Depression and anxiety disorders were the primary outcome variables. Diagnosis of depressive and anxiety disorders was made using the Structured Clinical Interview for DSM-5 (SCID-5), a semi-structured diagnostic interview designed to assess major DSM-5 psychiatric diagnoses [11]. The SCID-5 was administered by trained personnel to ensure consistency and reliability of diagnosis. The institution where the study was conducted has authorised access to DSM-5 diagnostic criteria and SCID-5 materials for clinical and academic use, and the tool was applied in accordance with its intended purpose.

Psychosocial factors assessed in the study included economic stress, work-related stress, duration of marriage, marital support, exposure to catastrophic or stressful life events, and perceived social support. These variables were included based on evidence from prior studies demonstrating their association with antenatal mental health disorders. Psychological factors were assessed using structured and semi-structured questions designed to identify clinically relevant symptoms and associated psychosocial stressors. The assessment included open-ended questions that allowed participants to describe their emotional state, mood changes, worries, fears, and stressors in their own words, followed by standardized, symptom-focused questions aligned with diagnostic criteria for depressive and anxiety disorders.

Sociodemographic data collected included age, education, and occupation of the respondent and her husband, income, address, and socioeconomic status as per the modified Kuppuswamy scale [12]. Obstetric history variables included gravidity and parity, last menstrual period, expected date of delivery, period of gestation, history of unplanned pregnancy, contraceptive use, antenatal complications or comorbidities such as gestational diabetes mellitus, gestational hypertension, and thyroid disorders, as well as details of the last childbirth. Additional variables included a history of postnatal depression or anxiety, childhood ailments, and a family history of mental disorders.

Physiological parameters such as height, weight, BMI, and haemoglobin levels were recorded to assess nutritional and health status, which may influence psychological outcomes during pregnancy.

Ethical considerations and confidentiality

The study was initiated only after obtaining approval from the Institutional Ethics Committee (RC/2023/137). All participants were informed about the purpose of the study, their right to refuse participation or withdraw at any stage, and the assurance that refusal would not affect the care they received. Confidentiality was strictly maintained by anonymizing the data and restricting access to the research team. No personal identifiers were used in data analysis or reporting.

Statistical analysis

All collected data were entered into Microsoft Excel (Microsoft® Corp., Redmond, WA) and subsequently analysed using the Statistical Package for Social Sciences (SPSS) version 23.0 (IBM Corp., Armonk, NY). Descriptive statistics were used to summarize the data, with categorical variables presented as frequencies and percentages and continuous variables expressed as means and standard deviations. Independent variables were categorized appropriately to examine their association with depression and anxiety disorders.

Logistic regression analysis was performed to assess the association between each independent variable and the outcome variables using odds ratios with 95% confidence intervals. The chi-square test was used to assess the statistical significance of associations. Variables showing significant or clinically relevant associations were considered for further analysis. A p-value of less than 0.05 was considered statistically significant for all analyses.

## Results

Most respondents were aged ≥25 years, 171 (95.0%), with a mean age of 26.8 ± 4.3 years. More than half of the women were graduates, 105 (58.3%), and the majority were homemakers, 154 (85.6%). Husbands were predominantly graduates, 106 (58.9%), or diploma holders, 61 (33.9%). Most participants were married for less than five years, 112 (62.2%), while 46 (25.6%) had been married for five to 10 years. The study population largely belonged to the upper-lower and lower-middle socioeconomic classes (Table 1).

**Table 1:** Sociodemographic characteristics of the study participants (N = 180)

Variable	Category	n (%)
Age (years)	<25	9 (5.0)
	≥25	171 (95.0)
	Mean ± SD	26.8 ± 4.3 years
Education (respondent)	Middle school	2 (1.1)
	High school	10 (5.6)
	Diploma	63 (35.0)
	Graduate	105 (58.3)
Occupation	Housewife	154 (85.6)
	Semi-skilled worker	3 (1.7)
	Skilled worker	23 (12.8)
Socioeconomic status (Kuppuswamy scale 2025)	Upper class	5 (2.8)
	Upper middle class	27 (15.0)
	Lower middle class	47 (26.1)
	Upper lower class	60 (33.3)
	Lower class	41 (22.8)
Education of husband	Middle school	1 (0.6)
	High school	12 (6.7)
	Diploma	61 (33.9)
	Graduate	106 (58.9)
Duration of marriage	<5 years	112 (62.2)
	5-10 years	46 (25.6)
	>10 years	22 (12.2)

Among the 180 pregnant women included in the study, primigravida and multigravida women were almost equally represented, accounting for 89 (49.4%) and 91 (50.6%) participants, respectively. The majority were in the third trimester of pregnancy (105, 58.3%), followed by the second trimester 63 (35.0%), while only 12 (6.7%) were in the first trimester. Unplanned pregnancies were reported by 24 (13.3%) women, whereas 156 (86.7%) had planned pregnancies. Comorbid medical conditions were present in 80 (44.4%) participants. Nearly half of the women had their last childbirth within the preceding two years 85(47.2%). With regard to nutritional status, 89 (49.4%) were overweight, and 52 (28.9%) were obese, while only 39 (21.7%) had a normal BMI. Anaemia was common, with 68 (37.8%) having mild anaemia and one (0.6%) having moderate anaemia; however, the majority had normal haemoglobin levels, 111 (61.7%) (Table 2).

**Table 2:** Obstetric profile of the study participants (N = 180)

Variable	Category	n (%)
Gravida	Primigravida	89 (49.4)
	Multigravida	91 (50.6)
Trimester	First	12 (6.7)
	Second	63 (35.0)
	Third	105 (58.3)
Unplanned pregnancy	Yes	24 (13.3)
	No	156 (86.7)
Comorbidities present	Yes	80 (44.4)
	No	100 (55.6)
Last childbirth ≤ 2 years	Yes	85 (47.2)
	No/none	95 (52.8)
BMI (WHO Classification)	Normal	39 (21.7)
	Overweight	89 (49.4)
	Obese	52 (28.9)
Haemoglobin (g/dL)	Moderate anaemia (7-8.99)	1 (0.6)
	Mild anaemia (9-10.99)	68 (37.8)
	Normal (≥11)	111 (61.7)

A notable proportion of the study participants reported psychosocial stressors. Work-related stress was present in 27 (15.0%) women, while economic stress was reported by 30 (16.7%). Marital discord was identified in 25 (13.9%) participants (Table 3).

**Table 3:** Psychosocial stressors among pregnant women (N = 180)

Psychosocial factor	Category	n (%)
Work-related stress	Yes	27 (15.0)
	No	153 (85.0)
Economic stress	Yes	30 (16.7)
	No	150 (83.3)
Marital discord	Present	25 (13.9)
	Absent	155 (86.1)

Among the 180 pregnant women assessed using SCID-5 criteria, depression was identified in 31 participants, yielding a prevalence of 17.2% (95% CI: 11.7%-22.7%), while anxiety was present in 14 participants, corresponding to a prevalence of 7.8% (95% CI: 3.9%-11.7%). The majority of women did not meet diagnostic criteria for depression (82.8%) or anxiety (92.2%). These findings indicate that depressive symptoms were more common than anxiety disorders in this population, although a clinically relevant proportion of women experienced mental health morbidity during pregnancy (Table 4).

**Table 4:** Prevalence of depression and anxiety among pregnant women (N = 180) SCID-5, Structured Clinical Interview for DSM-5

Condition	Category	n (%)	95% CI
Depression (SCID-5)	Present	31 (17.2)	11.7%-22.7%
	Absent	149 (82.8)	77.3%-88.3%
Anxiety (SCID-5)	Present	14 (7.8)	3.9%-11.7%
	Absent	166 (92.2)	88.3%-96.1%

Table 5 demonstrates several factors significantly associated with depression among pregnant women on both univariate and multivariable analysis. On unadjusted analysis, women aged <25 years had markedly higher odds of depression compared to those aged ≥25 years (OR = 11.7; 95% CI: 3.0-45.6), although this association lost statistical significance after adjustment (adjusted OR = 4.2; 95% CI: 0.9-19.2; p = 0.06). A duration of marriage greater than five years remained an independent predictor of depression (adjusted OR = 2.47; 95% CI: 1.10-5.55; p = 0.028). Unplanned pregnancy was also significantly associated with depression after adjustment (adjusted OR = 2.9; 95% CI: 1.1-7.8; p = 0.03). The presence of comorbidities showed a strong independent association, with women having comorbid conditions exhibiting nearly five-fold higher odds of depression (adjusted OR = 4.8; 95% CI: 1.7-13.6; p = 0.003). Psychosocial stressors emerged as the strongest predictors; economic stress was associated with almost ten times higher adjusted odds of depression (adjusted OR = 9.6; 95% CI: 3.6-25.4; p < 0.001), while work-related stress increased the odds more than eightfold (adjusted OR = 8.2; 95% CI: 3.1-21.6; p < 0.001). Marital discord demonstrated the highest independent association with depression, with an adjusted OR of 14.7 (95% CI: 4.9-43.8; p < 0.001).

**Table 5:** Univariate and multivariable analysis of factors associated with depression among pregnant women (N = 180) *p-value < 0.05 is statistically significant. ^†^Logistic regression analysis. ^#^Modified Kuppuswamy Scale 2025.

Variable	Category	Depression present n (%)	Unadjusted OR	95% CI	Adjusted OR^†^	95% CI	p-value
Age (years)	<25	6 (66.7)	11.7	3.0-45.6	4.2	0.9-19.2	0.06
	≥25	25 (14.6)	Reference	-	Reference	-	-
Education	Graduate	14 (13.3)	0.48	0.23-1.98	-	-	-
	≤Diploma	17 (22.4)	Reference	-	-	-	-
Occupation	Housewife	29 (18.8)	2.41	0.55-10.45	-	-	-
	Employed	2 (7.7)	Reference	-	-	-	-
Duration of marriage	>5 years	18 (26.1)	2.89	1.31-6.38	2.47	1.10-5.55	0.028*
	≤5 years	13 (11.6)	Reference	-	Reference	-	-
Socioeconomic status^#^	Upper	7 (13.5)	Reference	-	Reference	-	-
	Lower	24 (19.2)	1.5	0.6-3.8	-	-	-
Trimester	First	4 (33.3)	Reference	-	-	-	-
	Second	12 (19.0)	0.47	0.13-1.7	-	-	-
	Third	15 (14.3)	0.33	0.09-1.2	-	-	-
Unplanned pregnancy	No	22 (14.1)	Reference	-	Reference	-	-
	Yes	9 (37.5)	3.6	1.4-9.2	2.9	1.1-7.8	0.03*
Comorbidities	No	6 (6.0)	Reference	-	Reference	-	-
	Yes	25 (31.3)	7.1	2.7-18.9	4.8	1.7-13.6	0.003*
History of abortion	No	23 (14.7)	Reference	-	Reference	-	-
	≥1 abortion	8 (33.3)	2.9	1.1-7.7	2.1	0.8-6.1	0.14
Last childbirth ≤2 years	No/none	15 (15.8)	Reference	-	-	-	-
	Yes	16 (18.8)	1.2	0.5-2.8	-	-	-
BMI	Normal	10 (25.6)	Reference	-	-	-	-
	Overweight	14 (15.7)	0.54	0.22-1.3	-	-	-
	Obese	7 (13.5)	0.45	0.16-1.3	-	-	-
Haemoglobin	Normal	15 (13.5)	Reference	-	-	-	-
	Anaemia	16 (23.2)	1.9	0.9-4.2	-	-	-
Economic stress	No	11 (7.4)	Reference	-	Reference	-	-
	Yes	20 (64.5)	22.8	9.1-57.1	9.6	3.6-25.4	<0.001*
Work-related stress	No	13 (8.5)	Reference	-	Reference	-	-
	Yes	18 (66.7)	21.4	8.5-53.7	8.2	3.1-21.6	<0.001*
Marital discord	No	12 (7.7)	Reference	-	Reference	-	-
	Yes	19 (76.0)	38.0	13.6-106.3	14.7	4.9-43.8	<0.001*

Table 6 summarizes the univariate and multivariable analyses of factors associated with anxiety among pregnant women. On unadjusted analysis, unplanned pregnancy showed a very strong association with anxiety (OR = 26.5; 95% CI: 7.7-91.4), which remained highly significant after adjustment (adjusted OR = 9.8; 95% CI: 2.9-33.1; p < 0.001). Economic stress was another independent predictor, with women reporting economic stress having more than fivefold higher adjusted odds of anxiety (adjusted OR = 5.4; 95% CI: 1.6-18.3; p = 0.006). Marital discord was also significantly associated with anxiety after multivariable adjustment (adjusted OR = 3.4; 95% CI: 1.0-11.9; p = 0.049). Although several factors such as lower education (unadjusted OR = 0.31; 95% CI: 0.10-0.97), longer duration of marriage (unadjusted OR = 3.21; 95% CI: 1.01-10.17), recent childbirth within two years (unadjusted OR = 5.7; 95% CI: 1.2-26.7), anaemia (unadjusted OR = 6.8; 95% CI: 1.9-24.4), and work-related stress (unadjusted OR = 5.2; 95% CI: 1.7-16.1) were associated with anxiety on univariate analysis, these associations did not retain statistical significance in the adjusted model.

**Table 6:** Univariate and multivariable analysis of factors associated with anxiety among pregnant women (N = 180) *p-value < 0.05 is statistically significant. ^†^Logistic regression analysis.

Variable	Category	Anxiety present n (%)	Unadjusted OR	95% CI	Adjusted OR^†^	95% CI	p-value
Age (years)	<25	2 (22.2)	1.78	0.38-8.20	-	-	-
	≥25	12 (7.2)	Reference	-	Reference	-	-
Education	Graduate	4 (3.8)	0.31	0.10-0.97	0.036	0.11-1.18	0.091
	≤Diploma	10 (13.2)	Reference	-	Reference	-	-
Occupation	Housewife	13 (8.4)	1.86	0.24-14.32	-	-	-
	Employed	1 (3.8)	Reference	-	-	-	-
Duration of marriage	>5 years	9 (13.0)	3.21	1.01-10.17	0.078	0.89-9.36	0.089
	≤5 years	5 (4.5)	Reference	-	Reference	-	-
Socioeconomic status	Upper	2 (3.8)	Reference	-	-	-	-
	Lower	12 (9.6)	2.7	0.6-12.3	-	-	-
Unplanned pregnancy	No	4 (2.6)	Reference	-	Reference	-	-
	Yes	10 (41.7)	26.5	7.7-91.4	9.8	2.9-33.1	<0.001*
Comorbidities	No	4 (4.0)	Reference	-	Reference	-	-
	Yes	10 (12.5)	3.4	1.0-11.2	2.1	0.6-7.6	0.24
History of abortion	No	11 (7.1)	Reference	-	-	-	-
	≥1 abortion	3 (12.5)	1.9	0.5-7.4	-	-	-
Last childbirth ≤2 years	No/none	2 (2.3)	Reference	-	Reference	-	-
	Yes	10 (11.8)	5.7	1.2-26.7	3.2	0.7-14.5	0.12
BMI	Normal	1 (2.6)	Reference	-	Reference	-	-
	Overweight	9 (10.1)	4.2	0.5-34.6	2.9	0.3-25.9	0.36
	Obese	4 (7.7)	3.1	0.3-29.8	2.2	0.2-22.7	0.52
Haemoglobin	Normal	3 (2.7)	Reference	-	Reference	-	-
	Anaemia	11 (15.9)	6.8	1.9-24.4	2.9	0.8-10.6	0.11
Economic stress	No	5 (3.3)	Reference	-	Reference	-	-
	Yes	9 (30.0)	12.5	3.8-41.3	5.4	1.6-18.3	0.006*
Work-related stress	No	8 (5.2)	Reference	-	Reference	-	-
	Yes	6 (22.2)	5.2	1.7-16.1	2.8	0.8-9.6	0.11
Marital discord	No	8 (5.2)	Reference	-	Reference	-	-
	Yes	6 (24.0)	5.8	1.8-18.9	3.4	1.0-11.9	0.049*

## Discussion

The present study assessed the prevalence of prenatal depression and anxiety among pregnant women attending a tertiary care outpatient clinic and examined their association with a range of sociodemographic, obstetric, psychosocial, and physiological factors. Using a structured diagnostic approach based on the Structured Clinical Interview for DSM-5 (SCID-5), the prevalence of depression and anxiety was found to be 17.2% and 7.8%, respectively. These findings indicate that a substantial proportion of pregnant women experience clinically significant mental health disorders during pregnancy, underscoring the importance of antenatal mental health as a public health priority.

The prevalence observed in this study is comparatively lower than that reported in several Indian and international studies, yet it remains clinically meaningful. The use of a DSM-5-based diagnostic interview rather than a screening questionnaire likely contributed to the relatively lower prevalence estimates, as diagnostic tools are more specific and less prone to overestimation. In the present study, a cut-off score of ≥4 was used to identify depression and anxiety, which has been shown to yield high sensitivity (100%) and specificity (95%) in Indian settings [11]. The prevalence of antenatal depression observed here is comparable to findings from a study conducted in Kerala and Tamil Nadu, which reported a prevalence of 16.3% [13]. Other Indian studies have reported lower prevalence rates, such as 9.18% in Navi Mumbai and 12.3% in Bangalore [14,15]. Variations across studies may be attributed to differences in study settings, diagnostic tools, sample characteristics, and underlying sociodemographic and psychosocial contexts.

With respect to anxiety, the prevalence in the current study (7.8%) was lower than that reported in several international studies. A study from China reported a prevalence of perinatal anxiety of 17.4% [16], while studies from Saudi Arabia and Brazil reported prevalence rates of 23.6% and 26.8%, respectively [17,18]. The lower prevalence in the present study may reflect sociocultural differences, differences in healthcare access, and the use of diagnostic interviews rather than symptom-based screening scales. Additionally, the tertiary care setting, which often caters to women with better access to specialist care, may have influenced these estimates. Nevertheless, the presence of anxiety in nearly one in twelve pregnant women highlights the need for routine assessment and timely intervention.

In the present study, no significant association was observed between depression or anxiety and several sociodemographic variables, including the age, education, and occupation of the respondent, as well as the age, education, and occupation of the husband. This finding contrasts with some previous studies that have identified maternal age as an important determinant of antenatal depression and anxiety. For instance, Cena et al. reported a higher prevalence of depression among women aged 30-35 years [19]. Such discrepancies may reflect contextual differences in resource availability, family support systems, and sociocultural expectations, which can modify the impact of demographic factors on maternal mental health.

Obstetric factors emerged as important contributors to antenatal depression and anxiety in this study. The progression of pregnancy, presence of pregnancy-related comorbidities, history of previous abortion, and a short interpregnancy interval (last childbirth ≤2 years) were positively associated with depressive and anxiety disorders. These findings suggest that cumulative physical and emotional stress related to pregnancy and childbirth may exacerbate vulnerability to mental health disorders. The observed increase in prevalence with advancing gestational age, particularly during the third trimester, aligns with findings from a study in Thailand, which reported a high prevalence of antenatal depressive symptoms (46.8%) during late pregnancy [20]. Heightened physical discomfort, fear of childbirth, concerns about foetal health, and anticipation of parenting responsibilities may contribute to increased psychological distress during this period.

Pregnancy-related comorbidities such as gestational diabetes mellitus, gestational hypertension, preeclampsia, and foetal growth restriction were found to increase the risk of depression and anxiety, although not all women with these conditions experienced mental health disorders. The association between medical complications and psychological morbidity highlights the bidirectional relationship between physical and mental health during pregnancy. Similarly, a shorter interval since the last childbirth was associated with a higher risk, possibly due to increased caregiving burden, physical exhaustion, and limited time for psychological recovery.

In contrast, physiological parameters such as BMI and haemoglobin levels did not show a significant association with depression or anxiety in the present study. This finding differs from reports suggesting a higher prevalence of antenatal and postpartum mental disorders among women with obesity [21]. The lack of association in the current study may be related to sample size, differences in BMI categorization, or the presence of stronger psychosocial determinants that overshadow the influence of physiological factors.

Psychosocial factors were the strongest and most consistent predictors of both depression and anxiety in this study. Socioeconomic status, marital discord, and unplanned pregnancy demonstrated significant associations with antenatal mental health disorders. Women experiencing financial strain and unstable economic conditions, as given by socioeconomic status, were at substantially higher risk, emphasising the role of socioeconomic vulnerability in shaping mental health outcomes during pregnancy. Marital discord and strained relationships with spouses also emerged as critical risk factors, reflecting the importance of family dynamics and emotional support in the Indian sociocultural context [14].

Similar findings have been reported by Bedaso et al., who demonstrated a strong association between low support and increased risk of depression and anxiety during pregnancy [22]. These results reinforce the need to address psychosocial stressors as part of comprehensive antenatal care. The relationship between marital discord and antenatal mental health observed in this study is consistent with existing literature [20-22]. Women reporting increased interpersonal conflict had significantly higher odds of depression and anxiety. Emotional support from partners and family members plays a protective role by buffering stress and enhancing coping capacity during pregnancy. 

The study has several important strengths. First, it addresses a clinically and public health-relevant issue by estimating the prevalence of depression and anxiety among pregnant women, a group that is often under-screened despite well-documented maternal and foetal consequences. Second, the use of a tertiary care outpatient setting allowed inclusion of women with diverse obstetric and medical profiles, improving the clinical relevance and external validity of the findings for real-world antenatal care. Third, psychological disorders were assessed using a structured, clinician-administered diagnostic approach rather than relying solely on self-reported screening tools, enhancing diagnostic accuracy and reducing misclassification bias. Fourth, the study comprehensively examined multiple domains of risk, including sociodemographic, obstetric, psychosocial, and physiological factors, enabling a more nuanced understanding of determinants of perinatal mental health. 

This study has certain limitations that should be acknowledged. The cross-sectional design limits causal inference between identified risk factors and mental health outcomes. The study was conducted in a single tertiary care hospital, which may limit the generalizability of findings to community or primary care settings. The relatively short study period and reliance on self-reported psychosocial variables may have introduced reporting bias. Additionally, although SCID-5 provides robust diagnostic accuracy, the absence of longitudinal follow-up precludes assessment of the persistence or progression of mental health disorders into the postpartum period. Since this study was done in a tertiary care setup, it is expected that complex cases are reflected more. High comorbidity reported in this study tends to be an indicator of the same.

Despite these limitations, the findings have important clinical and public health implications. The study highlights that a considerable proportion of pregnant women experience depression and anxiety, with psychosocial stressors emerging as the most influential determinants. Routine screening for mental health disorders using standardized diagnostic tools, along with early identification of psychosocial risk factors, should be integrated into antenatal care services. Strengthening support systems, addressing marital and financial stressors, and providing timely mental health referrals may help mitigate adverse maternal and foetal outcomes. Integrating perinatal mental health services into existing maternal healthcare frameworks is essential for improving overall maternal and child health outcomes.

## Conclusions

This study demonstrates that prenatal depression and anxiety are prevalent among pregnant women attending a tertiary care outpatient clinic, with depression affecting 17.2% and anxiety affecting 7.8% of participants. Although sociodemographic factors showed no significant association, obstetric variables such as advancing gestational age, pregnancy-related comorbidities, history of abortion, and short interpregnancy interval contributed to increased vulnerability. Psychosocial stressors, particularly economic stress and marital discord, emerged as the strongest and most consistent predictors of both depression and anxiety, with work-related stress for depression alone. These findings underscore the need for routine screening for mental health disorders during antenatal care and highlight the importance of addressing psychosocial determinants alongside obstetric management to improve maternal and foetal outcomes.
